# Photothermal and Magnetic Actuation of Multimodal PNIPAM Hydrogel-Based Soft Robots

**DOI:** 10.3390/gels11090692

**Published:** 2025-09-01

**Authors:** Xiangyu Teng, Zhizheng Gao, Xuehao Feng, Shuliang Zhu, Wenguang Yang

**Affiliations:** School of Electromechanical and Automotive Engineering, Yantai University, Yantai 264005, China; 15166828596@163.com (X.T.); gaozhizheng2024@163.com (Z.G.); 17660278786@163.com (X.F.)

**Keywords:** soft robot, motion performance, light-activated, magnetically actuated

## Abstract

Soft robot motion performance has long been a core focus in scientific research. This study investigates the motion capabilities of soft robots constructed using poly(N-isopropylacrylamide) (PNIPAM) hydrogels, with key innovations in material design and functional enhancement. By optimizing the hydrogel formulation and incorporating molybdenum disulfide (MoS_2_) to endow it with photothermal response properties, the material achieves muscle-like controllable contraction and expansion deformation—a critical breakthrough in mimicking biological motion mechanics. Building on this material advancement, the research team developed a series of soft robotic prototypes to systematically explore the hydrogel’s motion characteristics. A flytrap-inspired soft robot demonstrates rapid opening–closing movements, replicating the swift responsiveness of natural carnivorous plants. For terrestrial locomotion, a hexapod crawling robot utilizes the photo-induced stretch-recovery mechanism of both horizontally configured and pre-bent feet to achieve stable directional propulsion. Most notably, a magnetically driven rolling robot integrates magnetic units to realize versatile multimodal movement: it achieves a stable rolling speed of 1.8 cm/s across flat surfaces and can surmount obstacles up to 1.5 times its own body size. This work not only validates the strong potential of PNIPAM hydrogel-based soft robots in executing complex motion tasks but also provides valuable new insights for the development of multimodal soft robotic systems, paving the way for future innovations in adaptive and bio-inspired robotics.

## 1. Introduction

Over billions of years of natural evolution, countless organisms have developed sophisticated locomotion mechanisms [1,2]. Their environmental adaptation strategies and complex action execution have long served as key sources of inspiration for designing artificial mechanical systems [3,4]. The Venus flytrap can swiftly close its leaves to capture insects in an instant [5,6]; sea stars achieve flexible seabed movement through coordinated motion of their multiple arms [7,8]; and tumbleweeds roll with wind assistance to accomplish long-distance migration. These biological locomotion patterns not only demonstrate remarkable environmental adaptability but also reveal the intrinsic relationship between structural design and movement capabilities.

Traditional rigid robots often lack sufficient flexibility and adaptability when operating in complex, dynamic environments, making it challenging to replicate the precise, gentle movements of living organisms [9,10]. In contrast, soft robots—constructed from compliant materials—possess exceptional deformability and environmental compatibility, emerging as a promising solution to this limitation [11,12]. Among various flexible materials, PNIPAM hydrogel has garnered significant attention due to its unique thermosensitive properties [13,14,15]. This hydrogel exhibits a Lower Critical Solution Temperature (LCST) of approximately 32 °C: above this temperature, it undergoes dehydration-induced shrinkage, while, below 32 °C, it absorbs water and expands. This muscle-like contraction–relaxation behavior endows it with inherent advantages for simulating biological movements [16,17].

However, relying solely on temperature to regulate PNIPAM hydrogel deformation hinders precise local control and remote manipulation, restricting its further application in soft robotics [18]. Light-driven technology, with its non-contact operation and remote precision control capabilities, offers an effective solution to this challenge [19,20]. Integrating light-driven mechanisms with PNIPAM hydrogel is therefore expected to enable accurate deformation control [21,22].

Building on this premise, this study combines PNIPAM hydrogel with MoS_2_ nanomaterials—known for their excellent photothermal conversion performance—to fabricate a composite hydrogel with photoresponsive properties [23]. Leveraging MoS_2_’s ability to convert light energy into heat under illumination, the temperature of the PNIPAM hydrogel can be indirectly regulated, thereby controlling its contraction and expansion [24,25]. Concurrently, digital light processing (DLP)-based 3D printing technology was employed to achieve precise fabrication of complex hydrogel structures [26,27].

This research designed and fabricated a series of biomimetic soft robots: a Venus flytrap-inspired robot that performs light-controlled opening/closing movements to simulate predatory behavior; a starfish-inspired hexapod robot that achieves directional locomotion through light-regulated foot deformation; and a tumbleweed-inspired rolling robot that utilizes magnetic field actuation to realize multimodal movements, including straight-line travel, controllable turning, and obstacle crossing. Through investigating the motion performance of these robots, this work aims to provide novel insights and methodologies for the design and application of soft robotic systems.

## 2. Results and Discussion

### 2.1. Study on the Properties of PNIPAM Hydrogel

**Figure 1:**
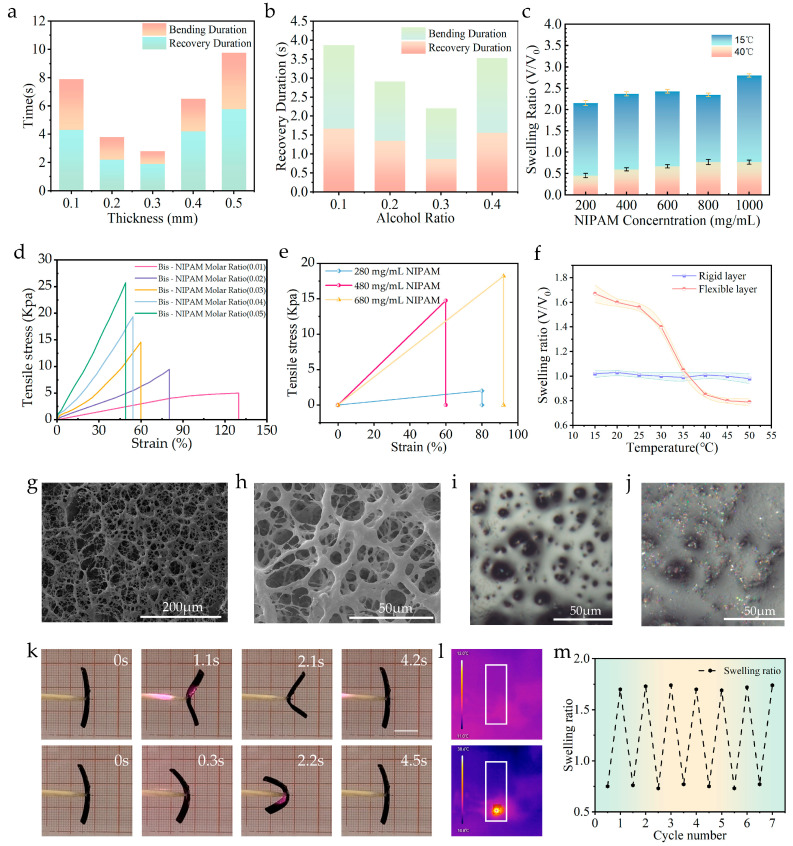
(**a**) Photoresponse time of PNIPAM hydrogels with different thicknesses. (**b**) Effect of alcohol content on the photoresponse performance of hydrogels. (**c**) Effect of NIPAM monomer content in hydrogel prepolymers on hydrogel properties. (**d**) The tensile stress–strain curves of PNIPAM hydrogel under different molar ratios of Bis and NIPAM. (**e**) Tensile properties of hydrogels at different monomer contents. (**f**) Deformation characteristics of rigid and flexible layers at different temperatures. (**g**,**h**) Scanning electron microscope (SEM) images of freeze-dried PNIPAM hydrogels under different fields of view. (**i**) Optical microscope image of PNIPAM hydrogel. (**j**) Optical microscope image of hydrogel after impregnation with MoS_2_. (**k**) Photoresponse experiment of hydrogel samples. (**l**) The temperature changes on the surface of the hydrogel as recorded by the thermal imager. (**m**) Cyclic durability test of hydrogels.

To optimize the performance of the hydrogel flexible layer, this study systematically examined how thickness, ethanol concentration, and N-isopropylacrylamide (NIPAM) content influence its photoresponsive deformation behavior and mechanical properties. Hydrogel thickness emerges as a critical parameter for regulating deformation characteristics and overall performance. In this work, hydrogel samples with thicknesses ranging from 0.1 mm to 0.3 mm were fabricated, and their bending deformation rates and recovery rates were precisely quantified post-illumination. Results showed that, for thicknesses below 0.3 mm, both bending rate and recovery rate increased with increasing thickness. However, when thickness exceeded 0.3 mm, the bending rate decreased significantly, accompanied by a reduction in maximum bending angle. This phenomenon indicates a negative correlation between photoresponsive rate and hydrogel thickness. Notably, while excessive thinning enhances photoresponsiveness, it causes significant deterioration in material strength and mechanical properties—such samples struggle to withstand large stresses and loads, thereby limiting practical application potential. Importantly, the 0.3 mm-thick sample exhibited excellent photoresponsive characteristics alongside balanced mechanical properties, meeting the manufacturing requirements for soft robotics (Figure 1a).

Ethanol concentration exerts a significant impact on hydrogel deformation behavior. Tests on samples with varying ethanol concentrations revealed that, within the 10–40% range, both bending deformation angle and recovery rate under near-infrared (NIR) light increased with ethanol concentration, peaking at 30%. Beyond 30% ethanol concentration, both bending deformation angle and light response rate showed a downward trend (Figure 1b). Under controlled conditions, hydrogel samples with NIPAM concentrations spanning 200–1000 mg/mL were prepared. Performance evaluations showed that reduced NIPAM content enhanced the hydrogel’s swelling capacity. Comprehensive assessment indicated that the sample with 480 mg/mL of NIPAM exhibited significant deformation capability, rapid response, and high strength—meeting the performance requirements for hydrogel robots (Figure 1c). Increasing bisphenol (Bis) content led to a significant enhancement in the hydrogel’s tensile modulus, confirming that Bis content is a critical regulator of hydrogel mechanical properties. This phenomenon can be attributed to changes in the microscopic cross-linking network structure within the hydrogel (Figure 1d). Further mechanical property tests on hydrogels with monomer concentrations of 280, 480, and 680 mg/mL demonstrated that the 480 mg/mL sample achieved the optimal balance between swelling behavior and mechanical performance (Figure 1e).

The directional bending of the hydrogel soft robot arises from the differential swelling behavior of its asymmetric bilayer structure, consisting of a flexible layer and a rigid layer. Specifically, the flexible layer requires significant swelling capacity, while the rigid layer exhibits minimal swelling. The shaded band region represents the error margin across repeated experimental measurements (Figure 1f). When the system absorbs water, the flexible layer undergoes substantial expansion, whereas the rigid layer volume remains relatively constant. This significant swelling discrepancy creates a strain gradient from the flexible layer to the rigid layer, inducing directional bending deformation of the entire structure.

Scanning electron microscopy (SEM) characterization showed that freeze-dried PNIPAM hydrogel possesses a highly porous network structure (Figure 1g,h), a feature further verified by optical microscopic imaging (Figure 1i). These porous characteristics significantly enhance MoS_2_ adsorption on the hydrogel matrix surface, with numerous MoS_2_ nanosheets uniformly anchored on pore surfaces (Figure 1j). This enhanced adsorption effectively improves light capture and photothermal conversion efficiency of the hydrogel system, thereby optimizing its photothermal response performance. MoS_2_-based composite materials demonstrate excellent stability across multiple photothermal cycles [28]. Moreover, the MoS_2_ used in this study remains stable under ambient conditions and exhibits no significant biotoxicity. Under NIR light irradiation, MoS_2_-modified hydrogel displayed distinct phototropic bending behavior (Figure 1k, Movie S1). To quantitatively characterize photothermal response behavior, thermal imaging technology was employed to monitor hydrogel surface temperature changes under NIR illumination. Experimental results showed that, after 1.2 s of illumination, the hydrogel surface temperature rapidly increased from an initial 12 °C to 38.6 °C—providing direct, robust experimental evidence for hydrogel phase transition and actuation behavior (Figure 1l). Cyclic actuation tests revealed that, after multiple NIR light-driven cycles, the hydrogel actuator retained its initial deformation capability and response speed with no significant performance degradation or plastic deformation, indicating excellent actuation durability. This confirms excellent compatibility and binding stability between the PNIPAM network and MoS_2_ nanosheets. Notably, a small amount of MoS_2_ nanosheets was released from the hydrogel network during the first few contraction/expansion cycles, after which the system stabilized with no further significant nanoparticle loss. To further assess stability under chemical stress, performance tests were conducted on the soft robot in acetic acid solution, where it successfully completed multiple opening–closing cycles. This confirms reliable chemical stability and functional retention of the material under acidic conditions (Figure 1m).

### 2.2. Motion Simulation of Venus Flytrap-Inspired Hydrogel Soft Robot

Inspired by the movement mechanism of the Venus flytrap, this study explores whether hydrogels can simulate such biological locomotion behaviors. Given that the geometric configuration of the flexible and rigid layers in the hydrogel structure plays a decisive role in bending deformation, we systematically designed various rigid layer morphologies and integrated each with the flexible layer to observe their deformation responses.

**Figure 2:**
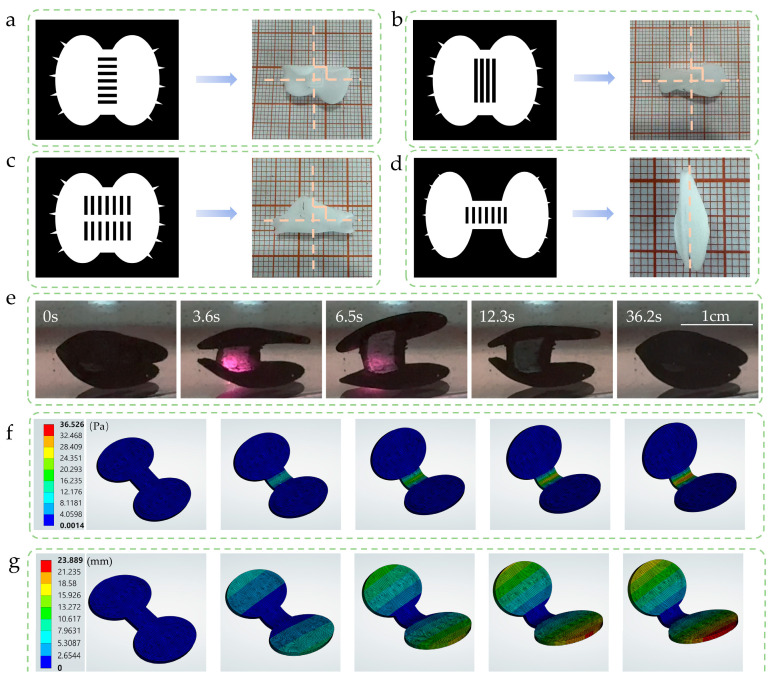
(**a**–**c**) Effects of different photomask patterns on the deformation of PNIPAM hydrogels. (**d**) Photomask pattern enabling the opening and closing movements of the Venus flytrap. (**e**) The flexible robot achieving opening and closing movements highly consistent with those of the Venus flytrap. (**f**) Stress simulation during the movement of the flexible robot. (**g**) Strain simulation during the movement of flexible robots.

In the initial configuration (Figure 2a), the flexible layer was designed to strictly replicate the shape of Venus flytrap leaves, with the rigid layer directionally bonded along the predefined bending axis. However, experimental results revealed a notable deformation deviation: the actual bending axis of the hydrogel was orthogonally aligned with the theoretical bending axis (angle ≈ 90°). To address this deviation, the second-stage experiment (Figure 2b) adjusted the geometric parameters and spatial arrangement of the rigid layer, yet results still exhibited the same orthogonal bending phenomenon. Further attempts to regulate deformation behavior via a dual-rigid-layer configuration also failed to achieve the expected movement effect (Figure 2c). Finally, by reconstructing the geometric shape of the flexible layer substrate and optimizing the rigid layer integration scheme (Figure 2d), we successfully realized biomimetic deformation behavior that highly matches the closing movement of the Venus flytrap. Analysis indicates that the key to simulating the typical bending deformation mode of Venus flytrap leaves lies in the design of the flexible layer configuration, which requires quantitative regulation of the length of deformation-producing key regions to precisely achieve the target deformation effect.

The fabricated soft robot was impregnated with MoS_2_ to endow it with photoresponsive capability. Without external stimulation, the soft robot maintains a normally closed initial state; under NIR light irradiation, it undergoes controllable opening deformation; and upon termination of irradiation, the system spontaneously returns to the closed state (Figure 2e, Movie S2). Further finite element analysis was employed to simulate the bending mechanical behavior of the device. The simulations were carried out using the Static Structural module in ANSYS Workbench2025R1. In the finite element model, the hydrogel material was simplified as a uniform, isotropic, hyperelastic material with a Young’s modulus of 25 kPa and a Poisson’s ratio of 0.45 to accurately represent the mechanical behavior of PNIPAM. Fixed constraints were applied at the bending axis to simulate the actual boundary conditions. The corresponding stress distribution and deformation fields were obtained by solving the model under these conditions. (Figure 2f,g). Results show uneven stress distribution during deformation, with significant stress concentration occurring in the primary bending axis region.

### 2.3. Driving Characteristics and Optimization of a Hexapod Soft Robot Based on Hydrogel Design

**Figure 3:**
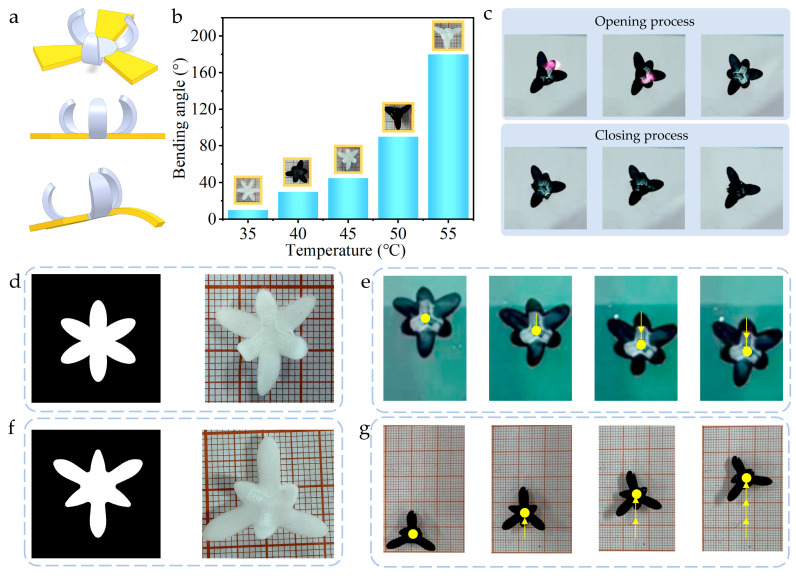
(**a**) Structure of the hexapod soft robot. (**b**) Effect of heating temperature on the bending angle of the hydrogel. (**c**) Photoresponse experiment of the three pre-bent legs. (**d**) Photomask pattern and physical structure of the soft robot driven by horizontal legs. (**e**) Directional movement of the soft robot pulled by horizontal legs. (**f**) Photomask pattern and physical structure of the soft robot driven by pre-bent legs. (**g**) Directional movement of the soft robot pulled by pre-bent legs. The arrow indicates the direction of movement.

Through rational structural design of hydrogels, soft robot movement can be driven by precisely controlling hydrogel deformation. Inspired by the locomotion mechanism of starfish, a soft robot featuring six leg structures was designed and fabricated (Figure 3a). To achieve directional propulsion, three of these legs were engineered as pre-bent legs. The key preparation process involves two critical steps: first, the hydrogel flexible layer undergoes heat-induced dehydration treatment to induce shrinkage, where the degree of shrinkage exhibits a positive correlation with heating temperature; second, bonding the rigid layer while the hydrogel remains in the contracted state enables the realization of directional bending. Experimental results demonstrate that the bending angle increases with rising preparation temperature (Figure 3b). The robot’s actuation relies on near-infrared (NIR) light stimulus response. When local NIR irradiation is applied to the three pre-bent legs, they respectively undergo reversible straightening deformation and revert to their bent state upon cessation of light exposure (Figure 3c, Movie S3). This controllable and reversible deformation characteristic serves as the core mechanism driving robot locomotion. The robot operates in two movement modes, utilizing either three horizontal legs or three pre-bent legs as driving units. In the horizontal leg driving mode, the adopted photomask pattern and its corresponding physical structure are illustrated in Figure 3d. By applying NIR light irradiation, the horizontal legs generate controllable bending deformation, thereby enabling directional movement of the soft robot (Figure 3e, Movie S4). However, when attempting to use pre-bent legs as the driving unit, experiments revealed that the soft robot failed to produce effective displacement. Preliminary analysis suggests this phenomenon may stem from a mismatch between the length of pre-bent legs and the driving force generated by the material, resulting in insufficient deformation-driven force to overcome the robot’s movement resistance. To optimize driving performance, targeted improvements were made to the photomask pattern, including significant shortening of the pre-bent leg design length, leading to the fabrication of a structurally optimized soft robot (Figure 3f). After optimization, under NIR light stimulation, the pre-bent legs exhibit forward stretching deformation; when light stimulation is removed, during the leg’s recovery to its initial bent state, the elastic restoring force successfully propels the soft robot forward (Figure 3g, Movie S5).

### 2.4. Rolling Soft Robot

#### 2.4.1. Design and Magnetic Field Driving Mechanism of Rolling Soft Robot

**Figure 4:**
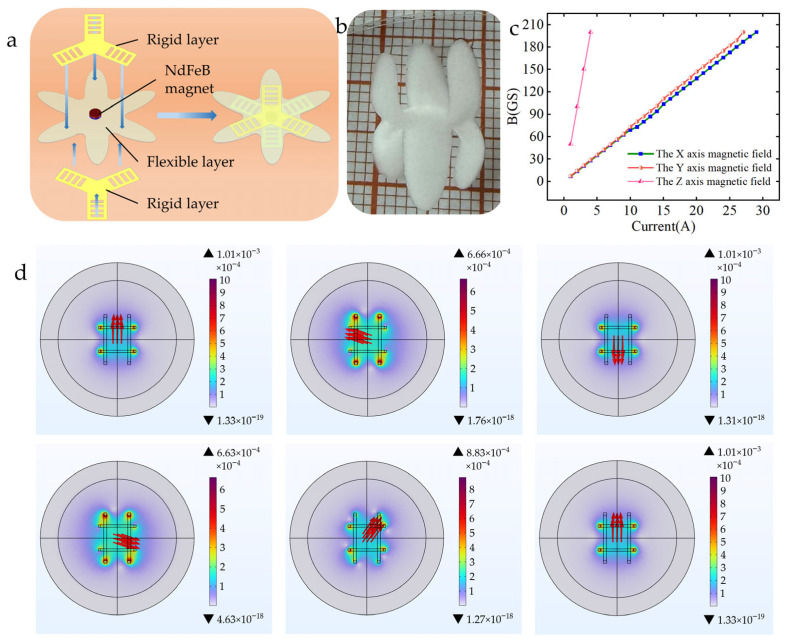
(**a**) Structural diagram of the rolling soft robot. (**b**) Physical image of the rolling soft robot. (**c**) Relationship between magnetic field intensity and current. (**d**) Simulation of the three-dimensional rotating magnetic field.

By integrating magnetically responsive units into the hydrogel matrix, the soft robot is endowed with controllable magnetic field-driven capabilities. Inspired by the rolling locomotion mechanism of tumbleweeds, a rollable soft robot was designed. Its core structure (Figure 4a) adopts a sandwich configuration: the middle layer consists of a flexible hydrogel layer, with rigid layers bonded to its upper and lower surfaces respectively. Magnetic units are embedded at the interface between the rigid layers and the flexible layer. This double-layer rigid structure design is intended to enable bidirectional symmetric bending deformation of the hydrogel, thereby significantly enhancing the robot’s obstacle-surmounting capability (Figure 4b). The robot is actuated by a controllable magnetic field generated by a Helmholtz coil system. Since the robot’s motion state is directly regulated by magnetic field intensity, the corresponding relationship between the output magnetic field intensity of the Helmholtz coil and the input current was first quantified (Figure 4c). To accurately analyze the robot’s dynamic behavior under a rotating magnetic field, an electromagnetic finite element model of the Helmholtz coil system was established. The key parameters of the model are set as follows: relative permeability of 1, relative dielectric constant of 1, conductivity of 6 × 10^7^ (S/m), and number of coil turns of 10. By applying sinusoidal alternating currents with a 270° phase difference to the orthogonal (XY-axis) coil pairs, the planar rotating magnetic field distribution required to drive the robot was successfully simulated (Figure 4d).

#### 2.4.2. Rolling Motion Characteristics and Dynamic Analysis of Soft Robots

**Figure 5:**
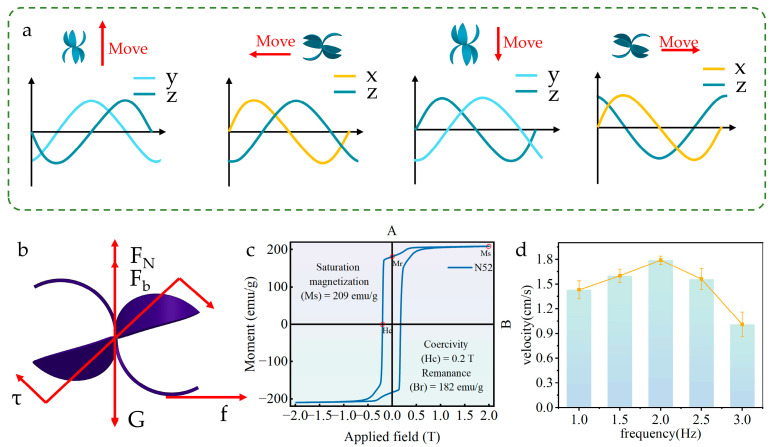
(**a**) Magnetic field waveforms driving the movement of the soft robot. (**b**) Force diagram of the soft robot. (**c**) Hysteresis loop of the neodymium magnet. (**d**) Speed variation in the soft robot at different frequencies.

Under the control of the electromagnetic actuation system (EMA), the soft robot can achieve multi-directional rolling motion, with a clear corresponding relationship between its rolling direction and the driving waveform (Figure 5a). The soft robot generates displacement through a rigid body-like rolling mode, and its stress distribution was analyzed (Figure 5b). Its locomotion capability stems from the internally integrated neodymium iron boron (NdFeB) permanent magnet components: these magnetic units enable the robot to exhibit free rolling behavior in response to an external rotating magnetic field, while the intrinsic properties of the magnetic material are characterized by a hysteresis loop (Figure 5c). When the robot is placed in the three-dimensional uniform magnetic field environment constructed by the Helmholtz coil system, its internal magnetic moment will orient itself under the action of the magnetic field, causing the long axis of the robot to align with the direction of the local magnetic field vector. The torque acting on the soft robot follows the formula:

(1) τ=m×B

where *m* is the magnetic moment of the magnet, and *B* is the magnitude of the magnetic field.

In addition, the translational motion of the soft robot is directly driven by the tangential friction force f at the contact interface, which realizes displacement output by converting rotational kinetic energy into translational kinetic energy:

(2) $$f=\frac{mB}{16\pi^{2}\mu r^{3}}$$

where μ is the fluid viscosity, and r is the radius of the micro-motor.

To further investigate its rolling dynamic characteristics, this study applied rotating magnetic fields with frequencies ranging from 1 Hz to 3 Hz via three groups of Helmholtz coils and systematically recorded the movement speed of the soft robot (Figure 5d). Experimental results show that, within the excitation frequency range of 1–2 Hz, the movement speed of the soft robot exhibits a positive correlation with the driving frequency. In this operational state, its maximum steady-state movement speed can reach approximately 1.8 cm·s^−1^. Under stable driving frequencies, the soft robot demonstrates consistent and predictable movement responses to magnetic field variations. When the driving frequency exceeds 2 Hz, the soft robot begins to exhibit motion instability, characterized by a significant decrease in speed. This instability mechanism is analogous to the slipping phenomenon occurring when traction between tires and the ground is insufficient during vehicle acceleration. In this high-frequency domain, achieving precise motion control of the soft robot becomes extremely challenging. Based on these findings, to ensure the accuracy and repeatability of motion control in experiments, this study set the upper limit of the driving frequency to 2 Hz. This frequency threshold selection effectively avoids motion instability while ensuring consistent speed responses of the soft robot under stable driving conditions, thereby significantly enhancing the reliability and repeatability of experimental data.

#### 2.4.3. Demonstration of Rolling Performance of Flexible Robot

**Figure 6:**
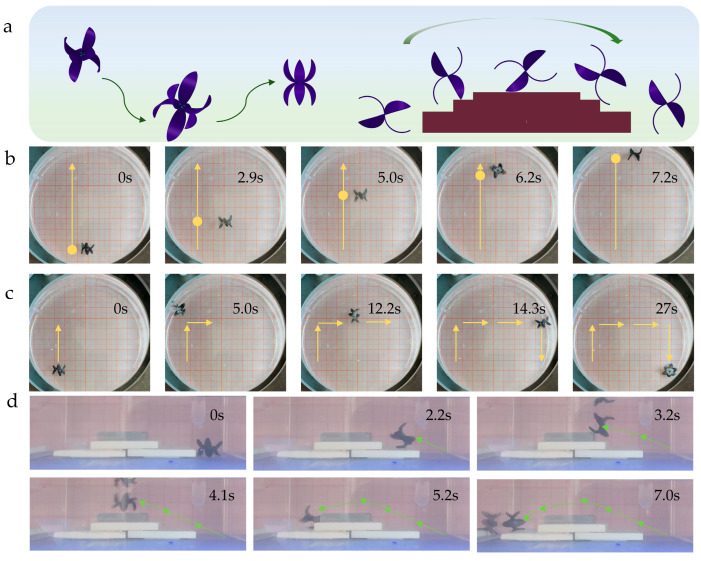
(**a**) Schematic diagram of the movement and obstacle-crossing of the rolling flexible robot. (**b**) Forward movement of the rolling soft robot. (**c**) Steering of the rolling soft robot. (**d**) Obstacle-crossing of the rolling soft robot. The arrow indicates the direction of movement.

The flexible robot designed in this study possesses multimodal movement capabilities, enabling it to stably perform various complex motion modes including linear travel, controllable steering, and obstacle crossing—fully reflecting its excellent movement adaptability and flexibility (Figure 6a). Experiments on the linear travel mode demonstrate that the robot exhibits highly stable performance in this movement state. Its movement trajectory shows distinct linear characteristics, thoroughly verifying the reliability and accuracy of its linear motion (Figure 6b, Movie S6).

In the verification of the controllable steering function, experiments recorded the robot’s complete operational process: it first performs an upward displacement to reach the edge of the Petri dish, then executes a precise right steering maneuver, radially crosses the entire Petri dish, and finally completes a downward displacement to reach the bottom of the Petri dish. The precise execution of this series of complex, directional three-stage trajectories is directly attributed to the accurate control of the external driving magnetic field (Figure 6c, Movie S7).

Furthermore, this study successfully conducted experiments on the flexible robot crossing stepped obstacles. Benefiting from its unique flexible structural design, the robot can effectively address the geometric challenges posed by obstacles and exhibit excellent obstacle-crossing performance. Notably, the height of the obstacles set in the experiments is approximately 1.5 times the robot’s own height. Under such highly challenging conditions, the robot can still successfully climb over, fully demonstrating its outstanding potential for overcoming high obstacles (Figure 6d, Movie S8).

### 2.5. Discussion

Compared with existing technologies in this field, the PNIPAM/MoS_2_ hydrogel actuator developed in this study exhibits a unique performance balance and multimodal driving advantages. In contrast to electroactive polymers (EAPs) that require kilovolt-level high voltage for actuation, this system achieves effective driving using low-power near-infrared light or magnetic fields, significantly enhancing biocompatibility and operational safety. Unlike pneumatic actuators that rely on pump-valve systems and face miniaturization challenges, this actuator realizes fully integrated built-in driving, making it more suitable for miniaturization and biological environment applications. Compared with certain molecular actuators that only function in specific chemical environments, this system demonstrates excellent robustness and adaptability in aqueous environments. Although it may not outperform specialized drivers in single performance metrics (such as absolute output force or speed), its core innovation lies in successfully integrating multiple light and magnetic driving modes while achieving comprehensive optimization in flexibility, safety, environmental adaptability, and wireless operation.

The current optical driving mode is primarily limited by photothermal diffusion effects and material response uniformity. While its motion control accuracy is inferior to that of electric field-based micro-driving systems, the magnetic driving component can achieve higher-precision trajectory control through magnetic field programming. Deviations mainly arise from environmental fluid resistance and contact interface friction. The 3D printing preparation process offers potential for microscale molding; further structural miniaturization can be achieved by optimizing optical paths and mask accuracy, though this will significantly increase preparation complexity. Moreover, as size decreases, fluid forces (such as viscous resistance) and surface effects will be significantly amplified, becoming key factors governing motion behavior.

Overall, the PNIPAM/MoS_2_ hydrogel actuator demonstrates significant advantages in integrating multiple driving methods and environmental adaptability, providing a promising solution for the application of soft robots in biomedicine and micro-operation fields.

## 3. Conclusions

This study systematically explored the motion performance of PNIPAM hydrogel-based soft robots. Through optimization of hydrogel preparation parameters, a hydrogel material with excellent light-responsive deformation capability, rapid recovery rate, and favorable mechanical properties was successfully developed. The hydrogel was functionally modified utilizing the photothermal properties of MoS_2_ nanoparticles, which significantly enhanced its light-harvesting and photothermal conversion efficiency, thereby endowing the material with stable and reversible light-driven deformation capabilities. Building on this foundation, a variety of biomimetic hydrogel soft robot structures were innovatively designed and fabricated. The Venus flytrap-inspired closing robot successfully achieved controllable light-driven opening–closing movements highly consistent with the biological prototype through precise regulation of the flexible layer substrate morphology and rigid layer integration scheme. The hexapod crawling robot achieved two modes of directional crawling via horizontal feet and pre-bent feet. For the magnetically driven rolling robot, integration of magnetic units into the hydrogel combined with a double rigid-layer structural design endowed it with excellent magnetic field actuation capability and obstacle-surmounting performance. Driven by a rotating magnetic field (≤2 Hz), it achieved stable, controllable multi-directional rolling (including straight movement and precise steering) with a maximum speed of 1.8 cm/s and could successfully surmount step obstacles equivalent to 1.5 times its own height.

In summary, this study not only deeply revealed the key performance parameters of PNIPAM hydrogels and their optimization strategies but also successfully realized efficient, controllable movements (including opening–closing, crawling, rolling, and obstacle-surmounting) in various biomimetic soft robots through ingenious structural design. The innovations of this research are prominently reflected in two aspects: materials and actuation structures. In the field of light-driven systems, MoS_2_ was employed as the photothermal conversion agent, replacing the more commonly used carbon nanotubes in this domain. MoS_2_ not only exhibits excellent photothermal conversion efficiency, but also its unique two-dimensional sheet structure facilitates better dispersion and anchoring within the hydrogel polymer network, significantly improving the uniformity and stability of the composite material. This provides a novel material solution for constructing high-performance light-driven hydrogel systems.

In the field of magnetic actuation, innovation lies in the unique structural design and actuation mode. Unlike most soft structures that encapsulate magnetic particles (typically exhibiting sliding or creeping motion), efficient rigid rolling motion was successfully achieved by embedding NdFeB permanent magnet units at a specific location (the interface between the rigid and flexible layers) and incorporating a symmetrical double rigid layer structure. Through collaborative innovation in materials and actuation modes, this work provides a novel design paradigm and experimental foundation for developing multimodal, high-performance soft robots capable of adapting to complex environments.

## 4. Materials and Methods

The solutes present in the PNIPAM hydrogel prepolymer solution include NIPAM monomer (98% purity, containing stabilizer MEHQ), Bis (99% purity), and photoinitiator TPO. NIPAM monomer and Bis were sourced from Shanghai Aladdin Biochemical Technology Co., Ltd. Shanghai, China, while TPO was acquired from Sigma-Aldrich. The water-dispersed photoinitiator nanoparticles contain 10% (*w*/*w*) of the type I photoinitiator diphenyl (2,4,6-trimethylbenzoyl) phosphine oxide (TPO). MoS_2_ (≥99.5% purity, 100 nm particle size) was purchased from MACKLIN.

**Figure 7:**
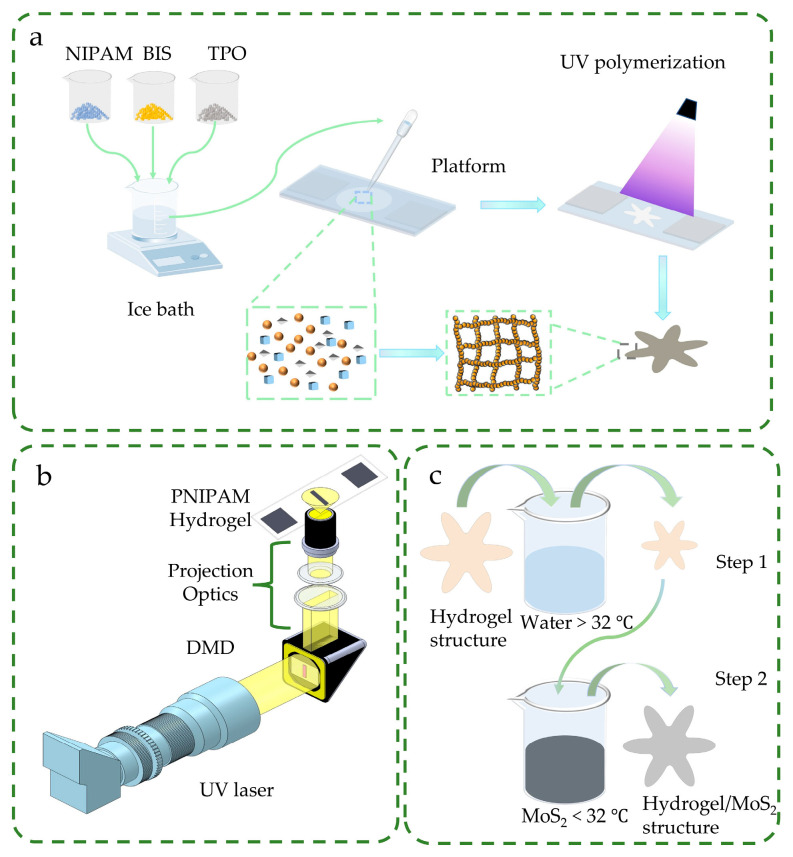
(**a**) Configuration process of hydrogel prepolymer. (**b**) Hydrogel printing platform. (**c**) Method of impregnating MoS_2_ into the hydrogel.

Preparation of PNIPAM hydrogel layers began with formulating the flexible layer prepolymer solution. Specifically, N-isopropylacrylamide (NIPAM; 480 mg), N,N′-methylenebis(acrylamide) (Bis; 13 mg), and diphenyl(2,4,6-trimethylbenzoyl)phosphine oxide (TPO; 12 mg) were accurately weighed into a 5 mL beaker. Anhydrous ethanol (300 μL) and deionized water (700 μL) were then added via pipette to form a homogeneous miscible solvent system (30% *v*/*v* ethanol). The mixture was dispersed and subjected to ultrasonic treatment for 10 min in an ice bath to obtain a homogeneous prepolymer solution. Subsequently, the rigid layer prepolymer solution was prepared using an analogous method, combining NIPAM (800 mg), Bis (30 mg), and TPO (12 mg) in the same solvent system (Figure 7a).

A custom digital light processing (DLP) printing system was employed in this study, consisting of four core subsystems: (1) a 405 nm ultraviolet (UV) light-emitting diode (LED) source optimized for photopolymerization; (2) a digital micromirror device (DMD) for pattern generation; (3) a projection optical path system utilizing THORLABS mirrors, lenses, and objective lenses to minimize pattern distortion during complex structure fabrication; and (4) an adjustable printing platform. The platform integrated a glass slide, a fluorinated ethylene propylene (FEP) film, and cover plates, with single-sided adhesive FEP film affixed to the two cover plates. The curing thickness was determined by the gap between the upper cover glass and lower FEP film, where each cover glass had a nominal thickness of 0.1 mm. To accommodate diverse experimental parameters, printing thickness was dynamically adjusted by varying the number of cover glass layers (Figure 7b). The 3D printing system operates on the principle of projecting a preset pattern onto the printing platform, inducing cross-linking and curing of the hydrogel prepolymer on the surface under UV light irradiation. The forming accuracy primarily depends on UV light exposure time: excessively long exposure may cause over-curing, reducing structural resolution, while insufficient exposure may result in inadequate cross-linking degree and weakened mechanical properties of the hydrogel. Therefore, systematic optimization of exposure time is required to balance high-precision structure and favorable mechanical properties. Additionally, to maintain printing consistency and hydrogel property stability, the prepolymer solution was replaced after every three printing operations to avoid adverse effects of monomer consumption and by-product accumulation on subsequent printing quality.

The fabrication of photothermally enhanced PNIPAM/MoS_2_ composites commenced with the preparation of a homogeneous MoS_2_ dispersion. This was achieved by magnetic stirring (25 min) of 500 mg MoS_2_ powder and 0.05 mL pre-dispersed MoS_2_ solution in 40 mL deionized water. Concurrently, PNIPAM hydrogel structures underwent cyclic thermal actuation: immersion in aqueous media above 32 °C (exceeding the LCST) induced dehydration-driven contraction, followed by rapid transfer to a <32 °C medium (for 45 s) to trigger rehydration expansion. During this expansion phase, capillary forces facilitated the infusion of MoS_2_ nanoparticles into the hydrogel network (Figure 7c). Iterative thermal cycling was continued until macroscopic darkening confirmed sufficient MoS_2_ incorporation, thereby yielding composites with optimized photothermal conversion performance.

As a photothermal conversion agent, the loading amount of MoS_2_ directly determines the light-reception efficiency and photothermal conversion capability of the material. Increasing the loading amount can enhance the thermal generation rate per unit time, causing the local temperature of the PNIPAM hydrogel to rise rapidly and exceed its LCST more quickly. However, excessively high MoS_2_ content tends to induce nanoparticle aggregation, which conversely reduces material uniformity and impairs mechanical properties. Therefore, in this study, the MoS_2_ loading amount was controlled at approximately 1.0 wt% to balance photothermal responsiveness and material stability.

On the other hand, light illumination intensity directly affects the energy input flux. Higher light intensity can enhance the photostimulation efficiency of MoS_2_, thereby generating higher thermal power density, accelerating temperature rise, and promoting faster deformation responses of the hydrogel. Notably, however, excessively high light intensity may cause local overheating and even irreversible thermal damage to the hydrogel network. In this study, the applied light intensity was approximately 1.2 W/cm^2^. This power density achieves rapid and significant temperature changes while remaining well below the threshold for inducing photothermal ablation of the material, thus maintaining material integrity while ensuring effective actuation.

## Data Availability

The data that support the findings of this study are available upon request from the corresponding authors.

## Supplementary Materials

The following supporting information can be downloaded at: https://www.mdpi.com/article/10.3390/gels11090692/s1, Movie S1: Photoresponse experiment of hydrogel samples; Movie S2: Soft robot achieve opening and closing movement; Movie S3: Photoresponse experiment of the three pre-bent legs; Movie S4: Directional movement of the soft robot pulled by horizontal legs; Movie S5: Directional movement of the soft robot pulled by pre-bent legs.; Movie S6: Forward movement of the rolling soft robot; Movie S7: Steering of the rolling soft robot.; Movie S8: Obstacle-crossing of the rolling soft robot.
